# Probiotic B420 and prebiotic polydextrose improve efficacy of antidiabetic drugs in mice

**DOI:** 10.1186/s13098-015-0075-7

**Published:** 2015-09-12

**Authors:** Lotta K. Stenman, Aurélie Waget, Céline Garret, François Briand, Rémy Burcelin, Thierry Sulpice, Sampo Lahtinen

**Affiliations:** DuPont Nutrition and Health, Active Nutrition, Sokeritehtaantie 20, 02460 Kantvik, Finland; Institut des Maladies Métaboliques et Cardiovasculaires de Rangueil, Rangueil Hospital, INSERM1048, 31432 Toulouse, France; Physiogenex SAS, Prologue Biotech, 516 Rue Pierre et Marie Curie, Labège Innopole, France

**Keywords:** Bifidobacteria, Diabetes, Gastroenterology, Obesity, Mice, Metformin, Probiotics, Prebiotics, Sitagliptin

## Abstract

**Background:**

Gut microbiota is now known to control glucose metabolism. Previous studies have shown that probiotics and prebiotics may improve glucose metabolism, but their effects have not been studied in combination with drug therapy. The aim of this study was to investigate whether probiotics and prebiotics combined with drug therapy affect diabetic outcomes.

**Methods:**

Two different study designs were used to test gut microbiota modulating treatments with metformin (MET) or sitagliptin (SITA) in male C57Bl/6J mice. In *Design 1,* diabetes was induced with four-week feeding with a ketogenic, 72 kcal% fat diet with virtually no carbohydrates. Mice were then randomly divided into four groups (n = 10 in each group): (1) vehicle, (2) *Bifidobacterium animalis* ssp. *lactis* 420 (B420) (10^9^ CFU/day), (3) MET (2 mg/mL in drinking water), or (4) MET + B420 (same doses as in the MET and B420 groups). After another 4 weeks, glucose metabolism was assessed with a glucose tolerance test. Fasting glucose, fasting insulin and HOMA-IR were also assessed. In *Design 2,* mice were fed the same 72 kcal% fat diet to induce diabetes, but they were simultaneously treated within their respective groups (n = 8 in each group): (1) non-diabetic healthy control, (2) vehicle, (3) SITA [3 mg/(kg*day)] (4) SITA with prebiotic polydextrose (PDX) (0.25 g/day), (5) SITA with B420 (10^9^ CFU/day), and (6) SITA + PDX + B420. Glucose metabolism was assessed at 4 weeks, and weight development was monitored for 6 weeks.

**Results:**

In *Design 1,* with low-dose metformin, mice treated with B420 had a significantly lower glycemic response (area under the curve) (factorial experiment, *P* = 0.002) and plasma glucose concentration (*P* = 0.02) compared to mice not treated with B420. In *Design 2*, SITA + PDX reduced glycaemia in the oral glucose tolerance test significantly more than SITA only (area under the curve reduced 28 %, *P* < 0.0001). In addition, B420, PDX or B420+PDX, together with SITA, further decreased fasting glucose concentrations compared to SITA only (−19.5, −40 and −49 %, respectively, *P* < 0.01 for each comparison). The effect of PDX may be due to its ability to increase portal vein GLP-1 concentrations together with SITA (*P* = 0.0001 compared to vehicle) whereas SITA alone had no statistically significant effect compared to vehicle (*P* = 0.14).

**Conclusions:**

This study proposes that combining probiotics and/or prebiotics with antidiabetic drugs improves glycemic control and insulin sensitivity in mice. Mechanisms could be related to incretin secretion.

**Electronic supplementary material:**

The online version of this article (doi:10.1186/s13098-015-0075-7) contains supplementary material, which is available to authorized users.

## Background

In recent years, the understanding of the link between gut microbiota and metabolic disease has rapidly increased. Various metabolic disorders have been linked to the gut microbiota through a common mechanism: metabolic endotoxaemia-induced inflammation [1]. In both animals [2–4] and humans [5] glucose metabolism disorders have been related to increased permeability of the gut barrier, which leads to increased translocation of endotoxins and even translocation of commensal bacteria [6]. These translocated bacteria form a so-called tissue microbiota [7], which evokes tissue inflammation [8] and, eventually, metabolic disorders [7, 9].

Since the first findings of a modified gut microbiota in obese mice and humans [10, 11], there has been an increasing recognition of the importance of gut microbial composition in metabolic health [12, 13]. Given the close relationship between the different components of metabolic syndrome, it has been difficult to pin-point specific species of bacteria that influence glucose metabolism or insulin resistance. Furthermore, methodological differences, high inter-individual variance and conflicting results have made it impossible to draw definitive conclusions on which species are altered [14]. However, decreased gut microbial diversity has been suggested to play a role in the development of metabolic disorders—a large study of approximately 300 participants showed that decreased gut microbial diversity is related to an unfavorable shift in the markers of glucose metabolism and low-grade inflammation [15]. When gut microbial diversity is improved with a dietary regimen, insulin sensitivity is also improved [16, 17]. Despite the lack of information regarding the specific gut microbial alterations impairing glucose metabolism, a landmark study using fecal transplantations presented preliminary but encouraging evidence of a causal relationship between gut microbes and glucose metabolism in humans [18]. Feces were transferred from healthy lean donors to male recipients with metabolic syndrome and, strikingly, the treatment improved the insulin sensitivity of the recipients.

The revelation of the involvement of gut microbiota has led to intense research on how to utilize this link to treat diabetes. Thus, several bacterial strains and dietary fibers have been tested for an effect on glucose metabolism [2, 17, 19–23]. We have previously shown that *Bifidobacterium animalis* ssp. *lactis* 420 improves insulin sensitivity and glucose tolerance while decreasing fat mass in dietary mouse models of diabetes and obesity [6, 24]. The treated mice also showed reduced tissue inflammation and endotoxaemia compared to the controls. Polydextrose, on the other hand, has been shown to induce satiation [25], ameliorate glycemic response [26] and reduce LDL cholesterol [27] in humans, pointing at a possible benefit in weight maintenance and metabolic health.

Currently, type 2 diabetes is primarily treated with drug therapy, such as the first line treatment metformin, with or without gliptins or sulfonylureas. Similar to all drugs, these treatments come with certain side effects. This fact is particularly true for metformin because approximately 30 % of patients must discontinue or dramatically reduce their daily therapeutic doses due to diarrheas. The dose reduction hampers the anti-diabetic efficacy of the treatment [28]. Dual treatments may be used to either improve the efficacy of the treatment or to reduce the dosage of an individual drug to prevent the occurrence of side effects.

The objective of this study was to test whether B420 and a dietary fiber, polydextrose (PDX) could improve the efficacy of metformin or gliptin in a mouse model of diabetes.

## Methods

### Animals and study designs

Male C57Bl/6J mice were obtained from Charles River (L’Arbresle, France) and acclimatized for at least 7 days prior to any experimentation. The mice were housed in groups of 4–6 animals per cage and maintained under a normal dark-light cycle (12 h/12 h), 22 ± 2 °C and 55 ± 10 % relative humidity. Tap water and feed were provided ad libitum. At 8–10 weeks of age, the mice were subjected to one of the two study designs outlined below.

*Design 1* First, we investigated the ability of B420 to potentiate the antidiabetic effect of metformin in diabetic mice. Metformin was used at a minimally active dose to ensure that the synergistic effect of the probiotic treatment on the antidiabetic drug could be tested. Diabetes was induced during a four-week feeding period with a ketogenic, 72 energy% fat diet, with 28 energy% protein and <1 energy% carbohydrate (UAR, France). This diet has been previously shown to increase fasting plasma glucose concentrations, impair glucose tolerance and lead to insulin resistance after only 4 weeks of feeding [29]. After the diabetes induction period, the mice were randomly allocated into groups. They were first ranked according to the value of the AUC of the intra-peritoneal glucose tolerance test (IPGTT) and then allocated sequentially to the different groups. This method ensures that all groups are characterized by a similar AUC of IPGTT. The values were confirmed by comparing body weight, IPGTT’s AUC, and plasma glucose and insulin concentrations. The average plasma glucose after the induction period was 9.18 ± 0.29 mmol/L, and there were no significant between-group differences. The experimental groups were as follows: (1) vehicle, diabetic controls with a daily gavage of saline; (2) B420, *Bifidobacterium animalis* ssp. *lactis* 420 (DuPont N&H), 10^9^ CFU gavaged daily; (3) MET, metformin (Sigma), 2 mg/mL in drinking water was used at a dose inducing minimal or no antidiabetic effect; and (4) MET + B420, 2 mg/mL metformin in drinking water and 10^9^ CFU B420 gavaged daily. There were ten mice in each group. The glucose metabolism parameters were measured after 4 weeks of treatment. The primary outcome was the AUC in the IPGTT test. The secondary outcomes were fasting plasma glucose, fasting plasma insulin and HOMA-IR (Homeostatic model assessment of insulin resistance) levels. HOMA-IR was calculated as: [Glucose] × [Insulin]/22.5.

*Design 2* Next, the effects of gut microbiota–modulating treatments combined with sitagliptin were studied in a prevention model to better distinguish between the vehicle and sitagliptin groups. The mice were first ranked according to their bodyweight and then randomly assigned into six groups; diabetes was induced simultaneously with the treatment regimens that were gavaged daily. In addition to B420, we now included a prebiotic, polydextrose (PDX), because of its promising results on satiety and metabolism in human studies [25–27]. A non-diabetic control group was added to better evaluate the magnitude of the effect, since our laboratory had less experience of the prevention model than the treatment model in *Design 1*, which we had developed ourselves. The following groups were established (n = 8 in each group): (1) NFD, healthy non-diabetic controls on a normal-fat diet; (2) vehicle, diabetic mice gavaged daily with saline; (3) SITA, sitagliptin, 3 mg/(kg*day); (4) SITA + PDX, sitagliptin with Litesse® Ultra polydextrose, 0.25 g/day; (5) SITA + B420, sitagliptin with B420 10^9^ CFU/day; and (6) SITA + PDX + B420 (as described above). Groups 2–6 continued on the ketogenic diet throughout the study. At 4 weeks, blood samples were drawn, and an IPGTT was performed. To monitor body weight and to reduce the effects of treatment and handling on the final samples, the mice were followed-up for two more weeks before being sacrificed. Ileum, pancreas and portal vein blood were harvested for further analysis. The primary outcome was the AUC in the IPGTT test. The secondary outcomes were fasting plasma glucose, fasting plasma insulin, HOMA-IR, portal vein GLP-1, ileal tissue GLP-1 and serum DPP-4 activity.

During both experimental designs, body weight was monitored weekly. All procedures were performed in accordance with the Guide for the Care and Use of Laboratory Animals (revised 1996) and French laws.

### Glucose tolerance tests

Glucose-tolerance tests were performed after four (Design 1) or six (Design 2) weeks of treatment to assess glucose management in mice. In Design 1 mice fasted for 6 h were injected with glucose (1 g/kg) into the peritoneal cavity to assess the overall antidiabetic effect of metformin. In Design 2, oral glucose tolerance tests (glucose 2 g/kg) were performed to assess the potentiation of the incretin effect of sitagliptin on glycaemia. The glucose response was followed from 30 min before the glucose challenge until 120 min after the challenge, measuring plasma glucose every 15–30 min using a standard glucose meter (Roche Diagnostics, Basel, Switzerland). The area under the curve was calculated from 0 to 120 min as the geometrical area between zero and the blood glucose curve for each individual mouse.

### Biochemistry

Blood samples for insulin assays were collected after 4 weeks of treatment from the tail veins of mice fasted for 6 h. Fasted plasma insulin was measured with an ELISA kit (#10-1247-01, Mercodia AB, Uppsala, Sweden).

In *Design* 2, ileum and portal blood were collected upon sacrifice from the mice under fed conditions. Plasma was collected in the presence of a DPP-4 inhibitor (0.1 mM diprotine A, Bachem, Switzerland), immediately centrifuged and the plasma separated and kept frozen at −80 °C. The plasma active GLP-1 concentration was measured with ELISA from 100 µl of plasma (GLP-1 active 7-36, Alpco, NH, USA). The lower limit of detection was between 0.5 and 1 pM. Ileum samples were lysed in buffer containing 70 % ethanol (v/v) and 0.1 M HCl. The extracts were diluted 1/10,000 to 1/100,000. GLP-1 concentrations were then measured with an ELISA kit (#43-GP1HU-E01, Eurobio, Courtaboeuf, France). DPP-4 was analyzed from portal blood with a luminescent protease assay (#G8350, Promega, Madison, Wisconsin, USA) in order to confirm the efficacy of SITA, which is a DPP-4 inhibitor.

### Statistical analysis

Data were analyzed using GraphPad Prism 6 (GraphPad Software Inc., California, USA) and R: A Language and Environment for Statistical Computing, version 3.1.2 (http://www.R-project.org/).

In *Design 1* the study was performed in a 2 × 2 setting with two factors: MET and B420. Normality and equality of group variances were first assessed with Shapiro–Wilk test and Brown-Forsythe test. If the data were found unsuitable for parametric analyses directly, a logarithmic, square root or inverse transformation was applied to all data of the same biomarker in order to analyze the data parametrically. The statistical analysis was then performed as a 2x2 factorial analysis that gives p-values for the main effects of B420, MET, and their interaction. For the IPGTT test, one value from the 60-min time point was missing in the B420 group and, as a conservative approach, the value from the 30-min time point was used to calculate the AUC.

In *Design 2* we applied the same data transformations as in *Design 1* for consistency. After the transformations all data were found suitable for parametric analyses. Most data were analyzed with one-way ANOVA. Supplemental data were analyzed with two-way ANOVA. If the global *P* was significant, Tukey’s HSD test was used to assess between-group differences. All data are expressed as the mean ± standard error of mean (SEM), and significances are two-sided. Differences were considered to be statistically significant when *P* < 0.05.

## Results

### Pro- and prebiotics improve glucose regulation during anti-diabetic treatment

**Fig. 1:**
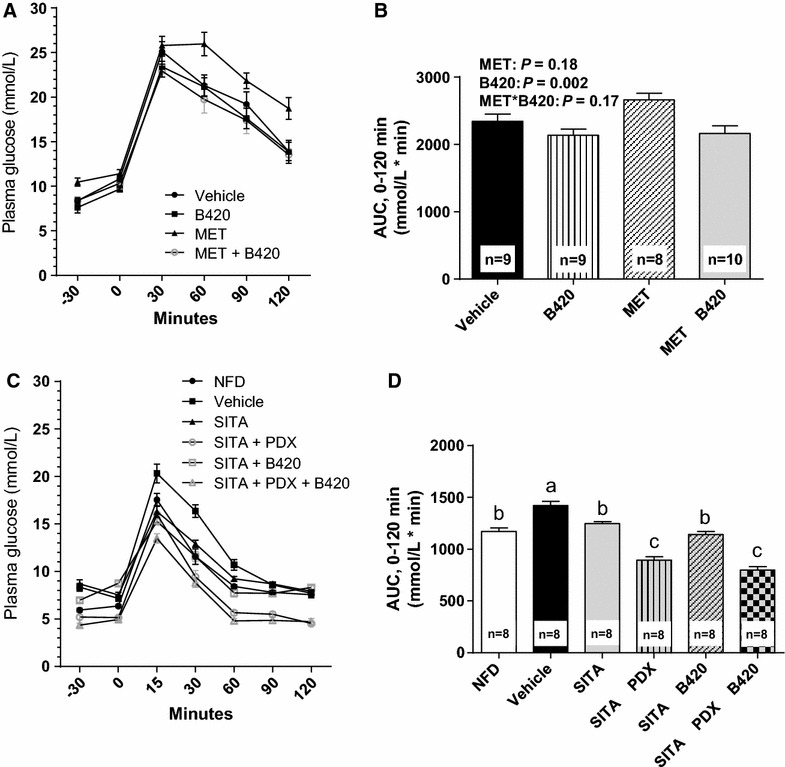
Glucose tolerance of mice after 4 weeks of treatment. Intraperitoneal glucose tolerance (**A**) and area under the curve (AUC) (**B**) of mice treated with metformin (MET) and/or *Bifidobacterium animalis* ssp. *lactis* 420 (B420) while on a high-fat diet. The oral glucose tolerance (**C**) and AUC (**D**) of mice treated with sitagliptin (SITA), polydextrose (PDX) and/or B420 while on a high-fat diet, in contrast to mice on a normal-fat diet (NFD). All data are expressed as the mean ± SEM. Groups without common* letters* differ significantly from one another (p < 0.05)

At the dose used in this design metformin demonstrated no effect on glucose tolerance in mice on a ketogenic diet (Fig. 1A, B) (*P* = 0.18). However, the B420-treated mice showed better glucose tolerance than those who were not treated with B420 (B420: AUC 2140 ± 93 mmol/L*min, MET + B420: 2160 ± 116 mmol/L*min, vehicle: 2340 ± 107 mmol/L*min, MET: 2660 ± 99 mmol/L*min, *P* = 0.002). No interactions were detected between MET and B420. Due to injection failures, IPGTT could not be performed to one mouse in the Vehicle group, two in the MET group and one in the B420 group.

In the sitagliptin design, the SITA mice demonstrated better glucose tolerance than the diabetic vehicle–mice (1250 ± 18 mmol/L*min vs. 1420 ± 42 mmol/L*min, *P* = 0.008) (Fig. 1C, D). The SITA + PDX and SITA + PDX + B420 mice showed further improved glucose tolerance compared to the SITA mice (894 ± 34 mmol/L*min and 798 ± 35 mmol/L*min for SITA + PDX and SITA + PDX + B420, respectively, both *P* < 0.0001 compared to SITA). SITA + B420 (1140 ± 28 mmol/L*min) showed no additional benefit compared to SITA (*P* = 0.24).

Body weight gain was also significantly reduced in the MET + B420 group compared to vehicle, as well as the SITA + PDX and SITA + PDX + B420 groups compared to SITA or vehicle (*P* < 0.05 for all comparisons) (Additional file 1: Figure S1). Metformin and sitagliptin alone had no effect on body weight.

### Fasting glucose, insulin and HOMA-IR

**Fig. 2:**
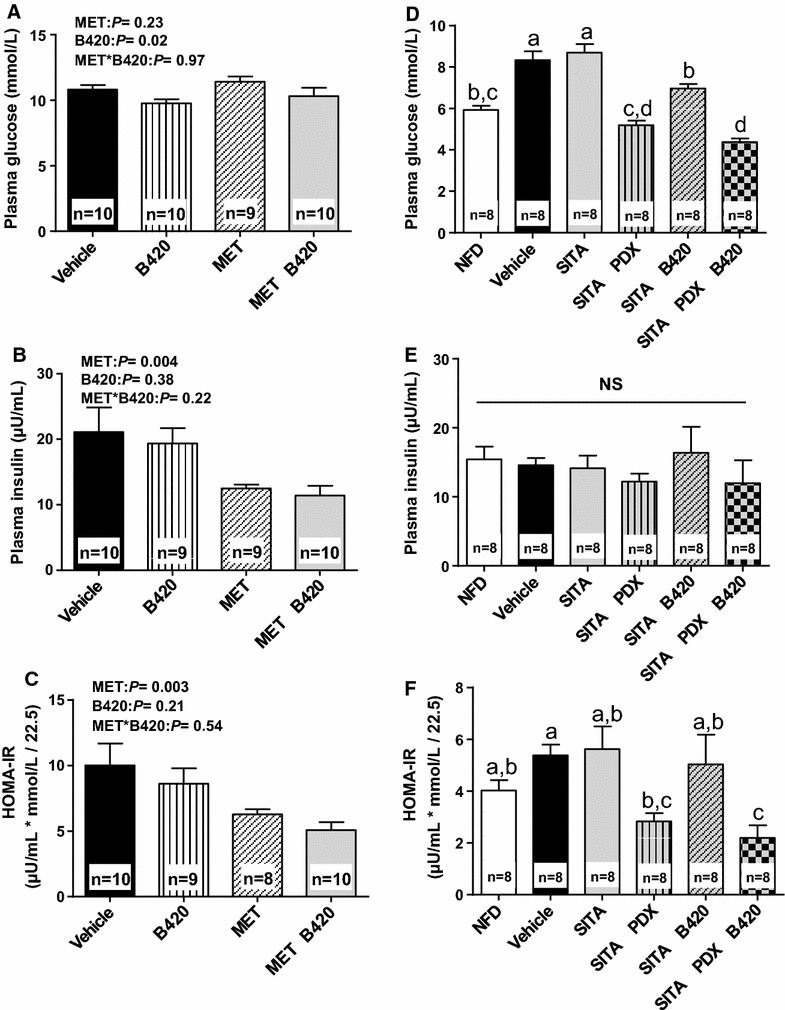
Fasted glucose (**A**, **D**), insulin (**B**, **E**) and HOMA-IR (**C**, **F**) after 4 weeks of treatment with metformin (MET), sitagliptin (SITA), polydextrose (PDX) and/or *Bifidobacterium animalis* ssp. *lactis* 420 (B420) in mice on a high-fat diet, in contrast to mice on a normal-fat diet (NFD). All data are expressed as the mean ± SEM. Groups without common *letters* differ significantly from one another (p < 0.05)

At this low dose, metformin had no significant effect on fasting glucose, but mice treated with MET had lower plasma insulin concentrations (MET: 12.5 ± 0.61 µU/mL, MET + B420: 11.4 ± 1.5 µU/mL, vehicle: 21.1 ± 3.8 µU/mL, B420: 19.4 ± 2.3 µU/mL, *P* = 0.004) and a lower HOMA-IR (MET: 6.27 ± 0.41 units, MET + B420: 5.07 ± 0.61 units, vehicle: 10.0 ± 1.7 units, B420: 8.61 ± 1.2 units, *P* = 0.003) than those who were not treated with MET (Fig. 2A–C). Mice treated with B420 had a significantly lower concentration of plasma glucose (B420: 9.77 ± 0.31 mmol/L, MET + B420: 10.3 ± 0.63 mmol/L, vehicle: 10.8 ± 0.33 mmol/L, MET: 11.4 ± 0.40 mmol/L, *P* = 0.02) than those not treated with B420, but there was no effect on plasma insulin or HOMA-IR. No interactions were detected between MET and B420. Due to technical difficulties, glucose could not be assessed from one mouse in the MET group, and insulin could not be assessed from one mouse in the B420 group and one in the MET group. These are reflected in the numbers of mice that could be included in the HOMA-IR calculations.

Sitagliptin had no significant effect on fasting glucose, insulin or HOMA-IR (Fig. 2D–F). However, all treatment groups with PDX and B420 showed lower fasting blood glucose than the vehicle and SITA groups (vehicle 8.3 ± 0.43 mmol/L, SITA 8.7 ± 0.42 mmol/L, SITA + PDX 5.2 ± 0.22 mmol/L, SITA + B420 7.0 ± 0.22 mmol/L, SITA + PDX + B420 4.4 ± 0.18 mmol/L, *P* < 0.01 for all SITA vs. treatments) (Fig. 2D). There were no differences between groups in fasting plasma insulin. However, HOMA-IR was significantly lower in the SITA + PDX + B420 group than both the vehicle and SITA groups (vehicle 5.4 ± 0.42, SITA 5.6 ± 3.5, SITA + PDX + B420 2.2 ± 0.49, *P* < 0.001 compared to both vehicle and SITA).

### GLP-1 release

**Fig. 3:**
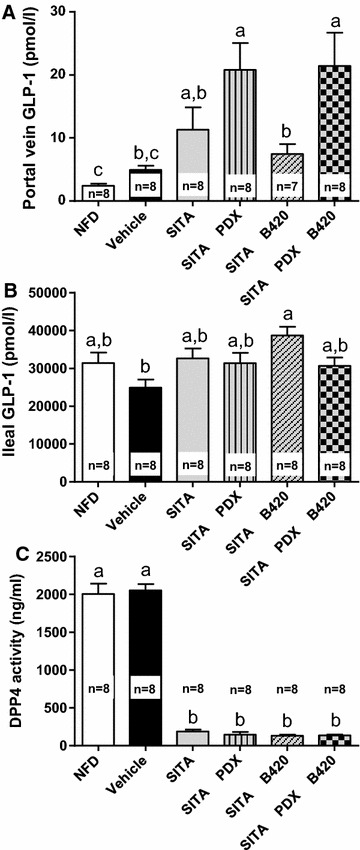
Portal vein (**A**) and ileal (**B**) GLP-1 concentrations and serum DPP-4 activity (**C**) after 6 weeks of treatment with sitagliptin (SITA), polydextrose (PDX) and/or *Bifidobacterium animalis* ssp. *lactis* 420 (B420) in mice on a high-fat diet, in contrast to mice on a normal-fat diet (NFD). All data are expressed as the mean ± SEM. Groups without common *letters* differ significantly from one another (p < 0.05)

To elucidate possible mechanisms, we investigated whether the selected gut microbiota modulators affect GLP-1 secretion from the gut; therefore, GLP-1 was measured from ileum and portal vein (Fig. 3A, B). The SITA + PDX and the SITA + PDX + B420 groups both showed a higher portal vein concentration of GLP-1 than the vehicle group (vehicle 4.9 ± 0.65 pmol/L, SITA + PDX 20.8 ± 4.3 pmol/L, SITA + PDX + B420 21.5 ± 5.3 pmol/L, *P* = 0.0001 for both groups vs. vehicle), whereas the SITA group did not significantly differ from vehicle (SITA 11.3 ± 3.6 pmol/L, *P* = 0.14 vs. Vehicle), nor did SITA + B420 (7.4 ± 1.6 pmol/L, *P* = 0.76). Due to technical issues, portal vein GLP-1 could not be measured from one mouse in the SITA + B420 group.

Ileal GLP-1 was not significantly increased by sitagliptin (vehicle 24,900 ± 2100 pmol/L, SITA 32,700 ± 2600 pmol/L, *P* = 0.26) (Fig. 3B). In contrast to what was observed for portal GLP-1 concentrations, only the SITA + B420 group had a higher level of ileum GLP-1 than the vehicle group (38,700 ± 2400 pmol/L, *P* = 0.006). None of the PDX treatment groups differed from vehicle.

To confirm efficacy of sitagliptin, we then measured the concentration of DPP-4 from portal vein. The SITA group had 91 % lower DPP-4 activity than the vehicle group, and activity was 93 % lower in the PDX and B420 treatment groups (*P* < 0.001 for all groups with SITA compared to NFD or vehicle). There were no statistically significant differences between the SITA groups.

## Discussion

In the present study, a potential probiotic B420 and/or Litesse® Ultra polydextrose were used together with metformin or sitagliptin to treat diabetes in mice. To our knowledge, this study is the first to demonstrate additional benefits for probiotic and prebiotic products on glucose metabolism when using antidiabetic drugs.

Metformin is used as the primary treatment for type 2 diabetes, and numerous specific mechanisms have been described. However, numerous mechanisms still remain to be uncovered. Metformin is known to increase the activation of AMP activated protein kinase (AMPK), an important enzyme for cellular energy homeostasis [30]. In the liver, AMPK activation leads to improved insulin receptor function and, thus, improved glucose transport, as well as reduced fatty acid synthesis. More recently, metformin has been shown to directly regulate gut microbiota metabolism; most notably, it affects the microbial methionine and folate metabolism [31]. The importance of metformin’s effect on gut microbiota was also demonstrated through its impact on the proliferation of *Akkermansia* [32]. In the latter case, when used as a prebiotic, metformin was shown to impact energy metabolism [33], and it was suggested that the beneficial effect of the antidiabetic drug does include the treatment of gut dysbiosis in type 2 diabetic patients [34]. Together, these molecular mechanisms lead to improved insulin sensitivity. In the present study we used a low dose of MET in order to see if MET combined with B420 could improve diabetes-related outcomes with a reduced risk of adverse effects. There were reduced plasma insulin concentrations and HOMA-IR levels in the groups treated with MET compared to those not treated with MET. B420 had no additional benefit on plasma insulin compared to metformin only, but B420 significantly attenuated the glucose response in IPGTT and decreased fasting plasma glucose concentration, suggesting that a combination treatment could be more effective in treating diabetes than metformin alone.

Sitagliptin, similar to all other gliptins, is an inhibitor of the enzyme DPP-4 a proconvertase that inactivates the incretins GLP-1 and gastric inhibitory peptide (GIP) by removing the first two N-terminal amino acid residues [35]. With this inactivation, sitagliptin increases the concentration of active incretins [36], which enhances glucose-induced insulin secretion and thereby improves oral glucose tolerance as reported in the present study. Only the groups treated with PDX showed further improved glucose tolerance compared to the SITA group. Sitagliptin had no effect on fasting glucose and insulin levels. Interestingly, however, B420 and PDX, as well as their combination, decreased the fasting concentration of plasma glucose compared to the vehicle and sitagliptin treatments. Together, B420 and PDX decreased HOMA-IR compared to sitagliptin only, although this effect seems to be purely due to the reduced fasting plasma glucose concentration.

As expected, all groups treated with SITA displayed minimal activity of DPP-4, the enzyme that SITA selectively inhibits. Consequently, SITA group had a slightly higher portal vein GLP-1 concentration compared to Vehicle, although the difference did not reach statistical significance.

Surprisingly, we showed that PDX promotes GLP-1 release into portal blood. GLP-1 treatment in humans decreases plasma glucose concentration without directly improving insulin sensitivity as assessed by hyperinsulinaemic clamp measurement [37]. Thus the impact of PDX on the glycemic control by sitagliptin could be mediated through GLP-1 release, which might be a plausible mechanism for the effect of PDX on fasting glucose.

B420 did not increase the portal blood GLP-1 concentration, but it did significantly increase the ileum GLP-1 concentration compared to the vehicle group, suggesting that whereas the tissue concentration of GLP-1 was enhanced by the probiotic, a further signal was perhaps required to trigger the peptide release into the circulation. The molecular mechanisms by which B420 could reduce glycaemia and fasting blood glucose concentrations could not be determined in this study. Nevertheless, several pieces of evidence indicate that B420 could improve gut barrier function [24, 38], and B420 is known to reduce tissue inflammation in mice on a high-fat diet [6, 24]. These key mechanisms control insulin resistance in type 2 diabetes and may explain the reduced fasting plasma glucose level demonstrated in this study. We recently showed that the combination of B420 and PDX normalized insulin resistance while preventing the high-fat diet-related decrease in Th17 and Treg cells in the ileal *lamina propria* [39].

The effect of B420 on glucose metabolism has been greater in previously published studies [6, 24]. Although these two previous studies were performed by the same research group, they were conducted at a different animal facility than the experiments in the present study. There are several factors that may cause differing results between laboratories, such as differences in the microbial environment, different animal handling practices, and factors related to the origin of the animals; note that the litter size of an individual mouse impacts its tendency to gain weight [40]. A small litter size results in so-called postnatal overfeeding, which results in obesity, insulin resistance, and glucose intolerance, which can even carry over to the offspring [41]. As long as these issues cannot be controlled between experiments, the exact replication of previously published study designs remains difficult.

In conclusion, both the probiotic and the prebiotic showed benefits to glycemic response and fasting plasma glucose but not fasting plasma insulin in mice. Whereas the low dose of metformin alone reduced plasma insulin concentration, the probiotic showed a complementary effect by lowering plasma glucose levels. Compared to sitagliptin only, polydextrose further decreased the oral glucose tolerance test response. In addition, fasting glucose was not affected by sitagliptin, whereas polydextrose, B420 and their combination all induced a significant decrease, although the polydextrose group showed the most marked improvement. The effect of polydextrose seems to be at least partly mediated through an increased concentration of portal GLP-1. The present study proposes a benefit for combining probiotics and/or prebiotics with antidiabetic drugs, this finding should be further assessed in clinical trials.

## Additional file

**Additional file 1: Figure S1.** Body weight gain in mice treated with metformin (MET) (A) or sitagliptin (SITA) (B) in combination with polydextrose (PDX) and/or *Bifidobacterium animalis* ssp. *lactis* 420 (B420) in mice on a high-fat diet, in contrast to mice on a normal-fat diet (NFD). All data are expressed as the mean ± SEM. Groups without common letters differ significantly from one another (p < 0.05). Ten mice per group are in panel A, and 8 per group are in panel B.

## Abbreviations

B420: *Bifidobacterium animalis ssp. lactis* 420
IPGTT: intraperitoneal glucose tolerance test
LPS: lipopolysaccharide
MET: metformin
NC: healthy non-diabetic control
PDX: polydextrose
