# Supplementary material for: Probiotic B420 and prebiotic polydextrose improve efficacy of antidiabetic drugs in mice
Source: Diabetol Metab Syndr. 2015 Sep 12;7:75. doi: 10.1186/s13098-015-0075-7 (PMC4567807; doi:10.1186/s13098-015-0075-7)
Supplement: Supplementary file 1 — Additional file 1: Figure S1. Body weight gain in mice treated with metformin (MET) (A) or sitagliptin (SITA) (B) in combination with polydextrose (PDX) and/or Bifidobacterium animalis ssp. lactis 420 (B420) in mice on a high-fat diet, in contrast to mice on a normal-fat diet (NFD). All data are expressed as the mean ± SEM. Groups without common letters differ significantly from one another (p < 0.05). Ten mice per group are in panel A, and 8 per group are in panel B. [file 13098_2015_75_MOESM1_ESM.pdf]

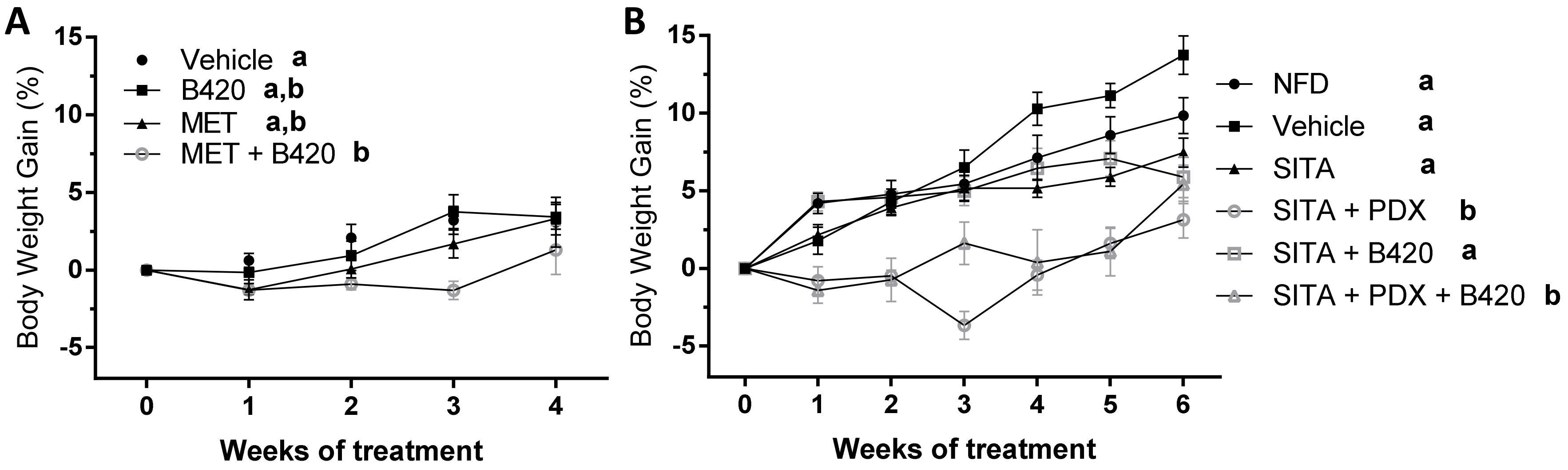

Supplemental Figure 1. Body weight gain in mice treated with metformin (MET) (A) or sitagliptin (SITA) (B) in combination with polydextrose (PDX) and/or *Bifidobacterium animalis* ssp. *lactis* 420 (B420) in mice on a high-fat diet, in contrast to mice on a normal-fat diet (NFD). Groups without common letters differ significantly from each other ($p < 0.05$). N = 8-10 per group.
